# A tale of two conditions: when people living with HIV meet three doses of inactivated COVID-19 vaccines

**DOI:** 10.3389/fimmu.2023.1174379

**Published:** 2023-06-19

**Authors:** Yuting Tan, Shi Zou, Fangzhao Ming, Songjie Wu, Wei Guo, Mengmeng Wu, Weiming Tang, Ke Liang

**Affiliations:** ^1^ Department of Infectious Diseases, Zhongnan Hospital of Wuhan University, Wuhan, China; ^2^ Wuhan Research Center for Infectious Diseases and Cancer, Chinese Academy of Medical Sciences, Wuhan, China; ^3^ Wuchang District Center for Disease Control and Prevention, Wuhan, China; ^4^ Department of Nosocomial Infection Management, Zhongnan Hospital of Wuhan University, Wuhan, China; ^5^ Department of Pathology, Zhongnan Hospital of Wuhan University, Wuhan, China; ^6^ Department of Pathology, School of Basic Medical Sciences, Wuhan University, Wuhan, China; ^7^ Institute for Healthcare Artificial Intelligence, Guangdong No.2 Provincial People's Hospital, Guangzhou, China; ^8^ The University of North Carolina at Chapel Hill Project-China, Guangzhou, China; ^9^ Hubei Engineering Center for Infectious Disease Prevention, Control and Treatment, Wuhan, China

**Keywords:** HIV, COVID-19, inactivated vaccine, booster dose, antibody response, T cell immunity

## Abstract

**Background:**

Currently, data on long-term immune responses to a homogenous booster dose of the inactivated COVID-19 vaccine are still limited among people living with HIV (PLWH).

**Methods:**

A prospective cohort study with a 13-month follow-up was conducted in China between March 2021 and August 2022 to evaluate the dynamics of SARS-CoV-2 specific humoral and cellular immunity against three doses of the inactivated COVID-19 vaccine from before the first dose until 6 months after the booster dose vaccination among PLWH in comparison to healthy controls (HC).

**Results:**

43 PLWH on antiretroviral therapy (ART) and 23 HC were enrolled. Compared with HC, the neutralizing antibodies (nAbs) levels among PLWH were significantly lower on days 14, 30, 60, 90, and 120 after the booster dose vaccination. Among PLWH, the nAbs titers on days 14, 30, and 60 after the booster dose were significantly higher than the peak of the second dose. However, on day 180 after the booster dose, the nAbs titers were similar to the peak of the second dose vaccination. Compared with HC, the frequencies of IFN-γ-secreting and TNF-α-secreting CD4^+^ and CD8^+^ T cells among PLWH were lower on days 14 and 180 after the booster dose vaccination. Among PLWH, increased T cell immunity was induced by the booster dose of the vaccine and kept stable on day 180 after the booster dose vaccination.

**Conclusion:**

Although a homogenous booster dose following two doses of the inactivated COVID-19 vaccine among PLWH could elicit higher nAb titers, reduce antibody decay, and maintain T cell responses even 6 months after vaccination, the overall immunogenicity of the booster dose was found to be lower among PLWH than among healthy controls. Further strategies are needed to improve immunogenicity to the inactivated COVID-19 vaccine among PLWH.

## Introduction

A high risk of COVID-19 mortality has been observed among people living with HIV infection (PLWH). Large-scale population-based retrospective cohort studies in South Africa and England reported that COVID-19 mortality increased 2~3-fold more in PLWH than in those without HIV infection (1, 2). Strengthening the prevention and control of SARS-CoV-2 infection in PWLH is crucial for global COVID-19 epidemic control.

COVID-19 vaccination is the primary strategy for preventing SARS-CoV-2 infection. The efficacy of two doses of COVID-19 inactivated vaccine for preventing symptomatic infections, hospitalizations, and deaths among healthy individuals has been verified by randomized clinical trials (3–5). In PLWH, persisting immunopathology may lead to impaired responses to vaccine despite virologic suppression (6). Our previous study indicated lower antibody responses against two doses of COVID-19 inactivated vaccine among PLWH than among healthy individuals (7, 8), and both our previous research and other studies observed decay in humoral immunity within 6 months after two doses of the inactivated or mRNA COVID-19 vaccination among people with immunosuppression, which may increase the risk of SARS-CoV-2 infection (9–11). Therefore, the booster vaccine is strongly proposed and expected to benefit PLWH.

Consistent with the results of previous studies on the booster the inactivated or mRNA COVID-19 vaccination in the general population (12, 13), we found that the level of neutralizing antibodies (nAbs) increased markedly and rapidly in PLWH on day 14 after a homogenous booster dose vaccination (14). However, data on the persistence of humoral and cellular immune responses following a homogenous booster dose of the inactivated COVID-19 vaccination among PLWH are still scarce. Here, we extend our previous research (8, 14) by conducting a prospective cohort study to investigate the longitudinal dynamics of the humoral and cellular responses of three doses of the inactivated COVID-19 vaccine among PLWH on stable antiretroviral therapy (ART) compared to healthy people.

## Methods

### Study participants

**Figure 1:**
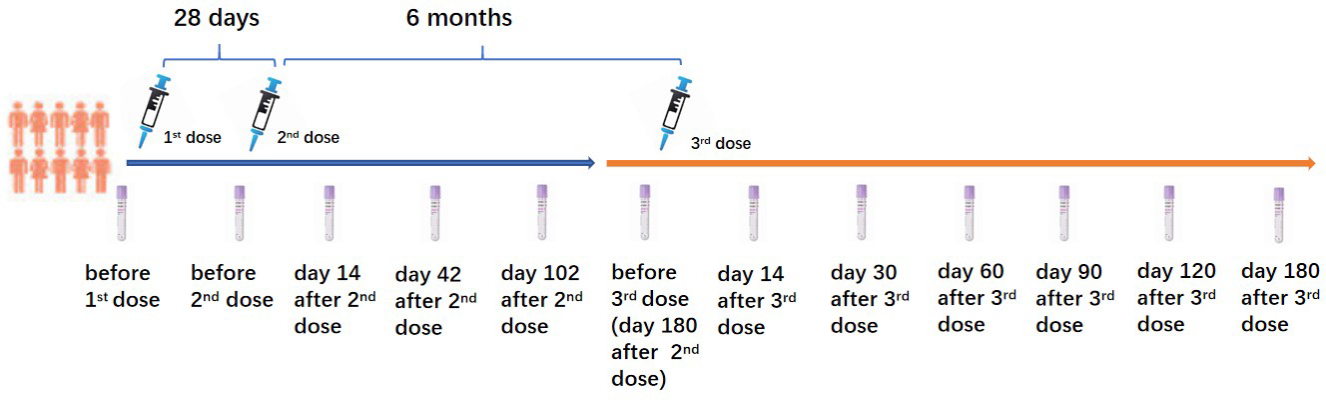
Three doses of inactivated COVID-19 vaccination schedule and sample collection.

This prospective cohort study was performed in Wuhan, Hubei Province, China, between March 2021 and August 2022. The 43 PLWH and 23 healthy controls (HC) who voluntarily participated in this study and received a homogenous booster dose of the inactivated COVID-19 vaccination were recruited from our previous study (8). All PLWH received stable ART for at least one year. PLWH and HC who had a history of SARS-CoV-2 infection confirmed by SARS-CoV-2 IgG, IgM tests, and a SARS-CoV-2 nucleic acid test before the first dose of the inactivated COVID-19 vaccination were excluded. All participants received two primary doses of the inactivated COVID-19 vaccine (Sinopharm, Wuhan Institute of Biological Products Co. Ltd) with an interval of 28 days. The homogenous booster dose of the vaccine (Sinopharm, Wuhan Institute of Biological Products Co. Ltd) was administered 6 months after the second dose vaccination. CD4^+^ T lymphocyte count (CD4 count) was tested in all PLWH before the first dose vaccination. The China National HIV/AIDS Comprehensive Response Information Management System (CRIMS) was used to acquire the clinical and laboratory data of PLWH. Peripheral blood of PLWH and HC were collected for the immunogenicity analyses at the following time points (as shown in **Figure 1**): 3 days before the first dose (D0B1); 3 days before the second dose (D0B2), on days 14 (D14A2), 42 (D42A2), 102 (D102A2) after the second dose; 3 days before the booster dose (D0B3), on days 14 (D14A3), 30 (D30A3), 60 (D60A3), 90 (D90A3), 120 (D120A3), and 180 (D180A3) after the booster dose vaccination.

### SARS-CoV-2 specific antibody responses examination

SARS-CoV-2 specific antibody responses of three doses of the inactivated vaccine were evaluated at all of the previously stated time points. The assessment of SARS-CoV-2 spike protein receptor-binding domain (RBD)-specific neutralizing antibodies (nAbs) was performed with in-house SARS-CoV-2 nAbs assay kits (Livzon, China). Semi-quantitative SARS-CoV-2 recombinant nucleocapsid (N) and RBD antigen-specific IgM and IgG antibodies were assessed with in-house-developed ELISA kits (Livzon, China). Seroconversion of nAbs was defined as the transition from baseline seronegative to seropositive or a 4-fold increase in antibody titers in seropositive participants at baseline (7).

### Isolation of PBMCs

Peripheral blood mononuclear cells (PBMCs) were isolated using the Ficoll-Paque method. Cell pellets were treated with 5 ml RBC lysis buffer (Sigma-Aldrich) for 10 min followed by washing once with 5% FBS-PBS. PBMCs were counted and cryopreserved with fetal calf serum (FCS) containing 10% dimethyl sulfoxide (DMSO) in liquid nitrogen.

### Intracellular cytokine staining and flow cytometry

SARS-CoV-2 specific CD4^+^ T and CD8^+^ T cell immunity against the inactivated COVID-19 vaccine were evaluated by intracellular cytokine staining (ICS) combined flow cytometry among PLWH and HC on day 14 after the second dose, before the booster dose, and on days 14, 30, and 180 after the booster dose vaccination. 1×10^6^ PBMCs were incubated overnight with SARS-CoV-2 Spike peptide pool (1mg/ml) containing 23 peptides from the wild-type SARS-CoV-2 virus in a 200μL final volume medium in round-bottom 96-well plates at 37°C and 5% CO2. Brefeldin A (10μg/ml) (GolgiPlug, BD Biosciences) was added in the last 5 hours of incubation. Surface marker staining was performed with anti-CD3-PerCP-cy5.5 (clone UCHT1; Biolegend), anti-CD8-APC-Cy7 (clone SK1; Biolegend), and anti-CD4-PE-Cy7 (clone RPA-T4; Biolegend) for 30 min at 4°C in the dark. Fixation and permeabilization solution (Cytofix/Cytoperm; BD Biosciences) was added for 45 min at 4°C in the dark. Intracellular cytokines staining with anti-IFN-γ-PE (clone 4S.B3; Biolegend) and anti-TNF-α-APC (clone MAb11; Biolegend) was then performed according to the manufacturer’s instructions. The stained cells were fixed with 2% paraformaldehyde and then analyzed by flow cytometry. Data were analyzed with FlowJo software version 10, and the gating strategy is shown in **Supplementary Figure 1**.

### Statistical analysis

SPSS 21.0 and GraphPad Prism 5.0 were used for data statistics and plotting. Variables were denoted as median (interquartile range, IQR) or proportion (%). The Kolmogorov-Smirnov test was used to analyze whether the measurement data conformed to the normal distribution. The group t-test was used to compare the continuous variables that conformed to the normal distribution, and the Mann-Whitney U test was used to compare continuous variables that weren’t normally distributed. The chi-square test or Fisher’s exact test was used to compare the categorical variables between the groups. p<0.05 was considered significant.

## Results

### Participant characteristics

The median age of PLWH and HC was 38 (IQR 33~45) and 33 (30~45) years old without significant difference between the two groups. The proportion of male participants in PLWH was 90.5%, significantly higher than that in HC (56.5%, p=0.008). A total of 39 (90.7%) PLWH had an HIV viral load under 50 copies/mL and reached virological suppression. The CD4 count had significant difference between PWLH and HC (p=0.015). The proportion of CD4 count over 500 cells/μl in HC (91.3%) was higher than that in PLWH (60.5%). The characteristics of 43 PLWH and 23 HC enrolled are shown in **Table 1**.

**Table 1:** Baseline characteristics of study participants.

	PLWH (n=43)	HC (n=23)	p-value
Age [years, median (IQR)]	38 (33~45)	33 (30~45)	0.196
Male, n(%)	38 (90.5)	13 (56.5)	0.008
Duration from diagnosis of HIV infection [years, median (IQR)]	7.2 (4.7~9.6)	/	/
Duration of Antiretroviral therapy [years, median (IQR)]	6.6 (4.3~8.1)	/	/
HIV-VL<50 copies/mL, n (%)	39 (90.7)	/	/
Baseline CD4 count (cells/μL, n(%) <200 200-500 >500	2 (4.6) 15 (34.9) 26 (60.5)	0 (0.0) 2 (8.7) 21 (91.3)	0.015

PLWH, people living with HIV infection; HC, healthy controls; CD4 count, CD4^+^ T lymphocyte count.

### Dynamics of nAbs against the inactivated COVID-19 vaccine

**Figure 2:**
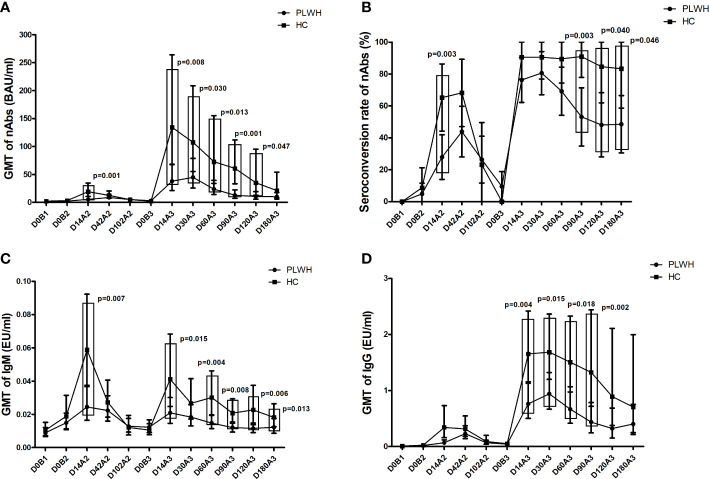
**(A)** The geometric mean titer (GMT) of SARS-CoV-2 specific neutralizing antibodies (nAbs) among PLWH and HC at multiple time points. **(B)** The seroconversion rate of SARS-CoV-2 specific nAbs among PLWH and HC at multiple time points. **(C)** The GMT of SARS-CoV-2 specific IgM among PLWH and HC at multiple time points. **(D)** The GMT of SARS-CoV-2 specific IgG among PLWH and HC at multiple time points. D0B1: Day 0 before the first dose; D0B2: Day 0 before the second dose; D14A2: Day 14 after the second dose; D42A2: Day 42 after the second dose; D102A2: Day 102 after the second dose; D0B3: Day 0 before the booster dose; D14A3: Day 14 after the booster dose; D30A3: Day 30 after the booster dose; D60A3: Day 60 after the booster dose; D90A3: Day 90 after the booster dose; D120A3: Day 120 after the booster dose; D180A3: Day 180 after the booster dose.

The dynamics of nAbs against three doses of the inactivated COVID-19 vaccine among PLWH and HC were evaluated. As shown in **Figure 2A**, in comparison to HC, the geometric mean titer (GMT) of nAbs in PLWH was significantly lower on day 14 after the second dose (p=0.001) and on days 14 (p=0.008), 30 (p=0.030), 60 (p=0.013), 90 (p=0.001), and 120 (p=0.047) after the booster dose vaccination. Furthermore, the nAbs of the second dose and the booster dose in PLWH peaked on day 42 and day 30 after vaccination, respectively, which were four weeks and two weeks behind the nAb peak appeared on day 14 after the second dose and the booster dose vaccination in HC.

Among PLWH, the nAbs titers on days 14 (p<0.001), 30 (p<0.001), and 60 (p=0.004) after the booster dose vaccination were significantly higher than the peak of nAbs titers of the second dose vaccination. Among HC, the nAbs titers on days 14 (p<0.001), 30 (p<0.001), 60 (p=0.002), and 90 (p=0.002) after the booster dose vaccination were significantly higher than the peak of nAbs titers of the second dose vaccination. In both PLWH and HC, the nAbs titers on day 180 after the booster dose were significantly higher than before the booster dose (i.e., on day 180 after the second dose, all p<0.001).

As shown in **Figure 2B**, the seroconversion rate of nAbs among HC on day 14 after the second dose (p=0.003) and on days 90 (p=0.003) and 120 (p=0.040) after the booster dose vaccination was significantly higher than that among PLWH. Among PLWH, the highest seroconversion rate of nAbs following the second and booster dose vaccination occurred on days 42 and 30 after vaccination, respectively. The latter was significantly higher than the former (80.6% vs. 43.9%, p=0.001). Among HC, the highest seroconversion rate of nAbs following the second and booster dose vaccination occurred on days 42 and 90 after vaccination, respectively, and there was no significant difference between them (90.9% vs. 68.2%, p=0.066).

### Dynamics of IgM and IgG against the inactivated COVID-19 vaccine

The GMT of SARS-CoV-2 specific IgM on day 14 after the second dose (p=0.007) and on days 14 (p=0.015), 60 (p=0.004), 90 (p=0.008), 120 (p=0.006), and 180 (p=0.013) after the booster dose vaccination among PLWH was significantly lower than that among HC (shown in **Figure 2C**). The peak of IgM titers of the booster dose was less than that of the second dose in both PWLH and HC. However, there were no statistically significant differences.

The GMT of SARS-CoV-2 specific IgG on days 14 (p=0.004), 30 (p=0.015), 60 (p=0.018), and 90 (p=0.002) after the booster dose vaccination among PLWH was significantly lower than that among HC (shown in **Figure 2D**). The peak of IgG titers of the booster dose was significantly higher than that of the second dose in both PWLH and HC (all p<0.001).

### T cell immune responses against the inactivated COVID-19 vaccine

**Figure 3:**
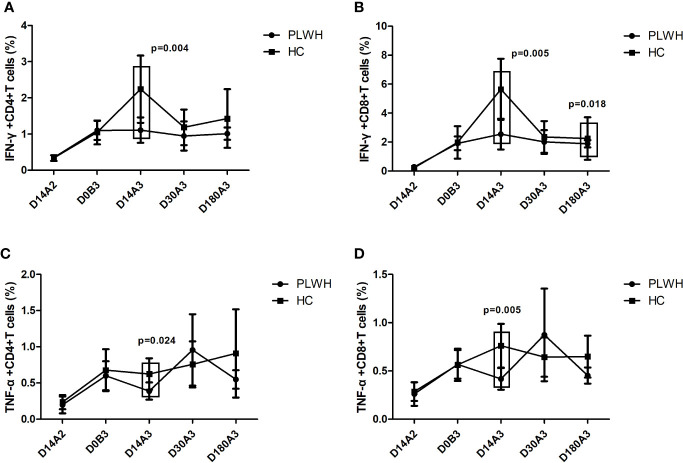
**(A)** The frequency of IFN-γ^+^CD4^+^T cells among PLWH and HC at multiple time points. **(B)** The frequency of IFN-γ^+^CD8^+^T cells among PLWH and HC at multiple time points. **(C)** The frequency of TNF-α^+^CD4^+^T cells among PLWH and HC at multiple time points. **(D)** The frequency of TNF-α^+^CD8^+^T cells among PLWH and HC at multiple time points. D14A2: Day 14 after the second dose; D0B3: Day 0 before the booster dose; D14A3: Day 14 after the booster dose; D30A3: Day 30 after the booster dose; D180A3: Day 180 after the booster dose.

For the comparison of PLWH and HC, as shown in **Figure 3**, the frequencies of IFN-γ^+^CD4^+^, IFN-γ^+^CD8^+^, TNF-α^+^CD4^+^, and TNF-α^+^CD8^+^ T cells among HC were significantly higher than that among PLWH on day 14 after the booster dose vaccination (p=0.004; p=0.005; p=0.024; p=0.005). The frequency of IFN-γ^+^CD8^+^ T cells in HC was significantly higher than in PLWH on day 180 after the booster dose vaccination (p=0.018).

**Figure 4:**
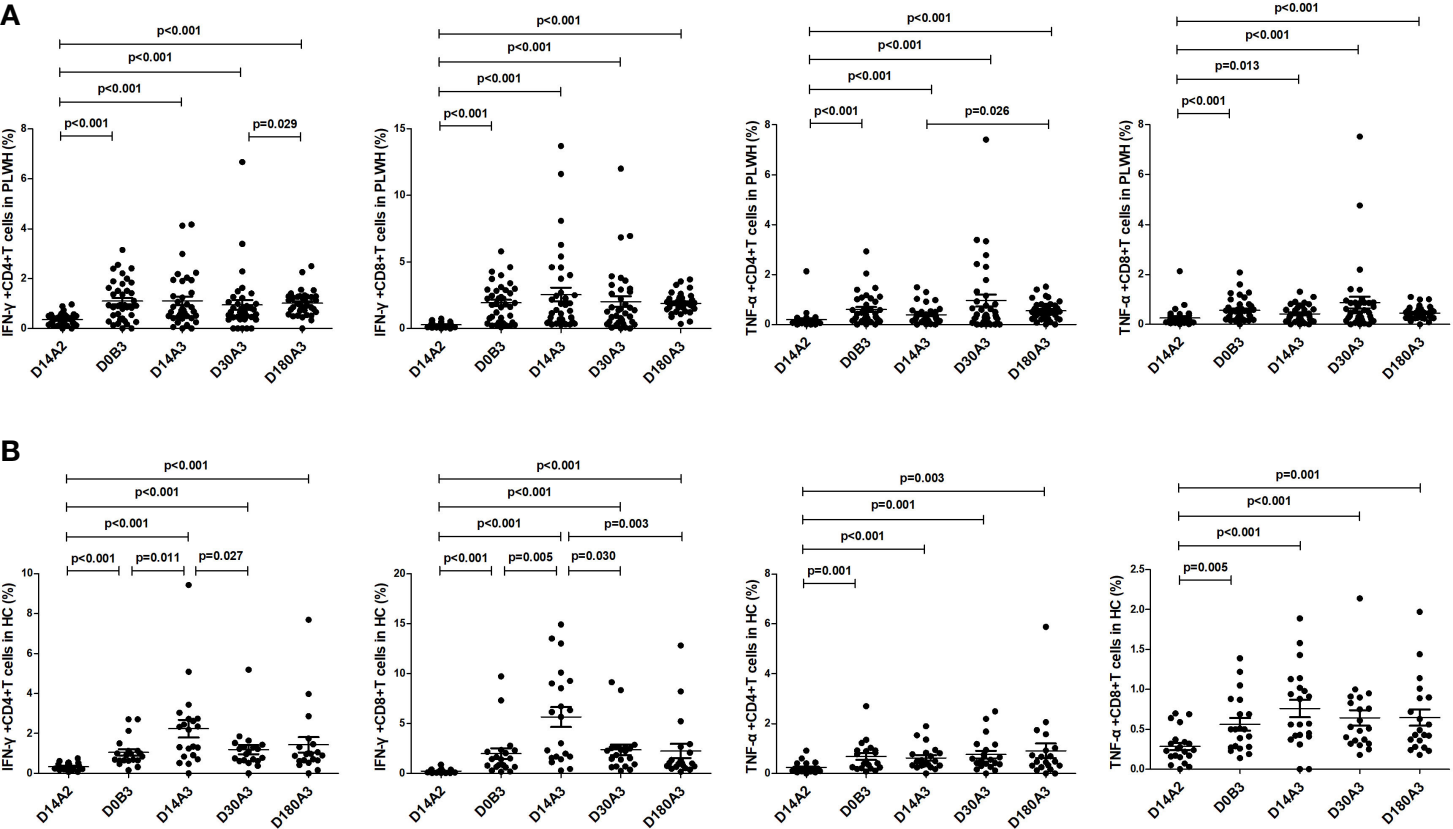
**(A)** The frequencies of IFN-γ-secreting and TNF-α-secreting CD4^+^ and CD8^+^ T cells in PLWH at multiple time points. **(B)** The frequencies of IFN-γ-secreting and TNF-α-secreting CD4^+^ and CD8^+^ T cells in HC at multiple time points. D14A2: Day 14 after the second dose; D0B3: Day 0 before the booster dose; D14A3: Day 14 after the booster dose; D30A3: Day 30 after the booster dose; D180A3: Day 180 after the booster dose.

As shown in **Figure 4**, among PLWH, the frequencies of IFN-γ^+^CD4^+^, IFN-γ^+^CD8^+^, TNF-α^+^CD4^+^, and TNF-α^+^CD8^+^ T cells on day 180 after the second dose (all p<0.001) and on days 14 (p<0.001; p<0.001; p<0.001; p=0.013), 30 (all p<0.001), and 180 (all p<0.001) after the booster dose were significantly higher than those on day 14 after the second dose vaccination. Furthermore, the frequencies of IFN-γ-secreting and TNF-α-secreting CD4^+^ and CD8^+^ T cells on day 180 after the booster dose were similar to those on day 180 after the second dose vaccination. Among HC, the frequencies of IFN-γ^+^CD4^+^, IFN-γ^+^CD8^+^, TNF-α^+^CD4^+^, and TNF-α^+^ CD8^+^ T cells before the booster dose (p<0.001; p<0.001; p=0.001; p=0.005) and on days 14 (all p<0.001), 30 (p<0.001; p<0.001; p=0.001; p<0.001) and 180 (p<0.001; p<0.001; p=0.003; p=0.001) after the booster dose were significantly higher than those on day 14 after the second dose vaccination.

## Discussion

Monitoring the immune responses of PLWH to the booster dose of the COVID-19 vaccine is essential for planning future COVID-19 measures. Our previous study investigated the humoral immune responses to two doses of the inactivated COVID-19 vaccine within 6 months after vaccination among PLWH (8). However, little is known about the longitudinal humoral and T cell immunity against the booster dose of the inactivated COVID-19 vaccine among PLWH. Hence, we extended the existing literature by reporting the longitudinal dynamics of humoral and cellular responses within 6 months after the booster dose of the inactivated COVID-19 vaccination among PLWH. We found that among PLWH, although a homologous booster dose of the inactivated vaccine could dramatically increase the nAbs level and maintain stable T cell responses, immunogenicity was still significantly lower than among HC at multiple time points.

In our present study compared with two primary doses of the inactivated vaccine, the booster dose of the inactivated vaccine could induce a rapid and stronger nAbs level and seroconversion rate and increase T cell immunity among PLWH in the early period. These results align with the previous study’s result, which showed that the third dose of mRNA vaccine could elicit quick, robust antibody responses among PLWH (15). In healthy individuals, a homologous booster dose of the inactivated vaccine has been reported to rapidly boost the nAb level 3~5-fold compared to the second dose (16, 17). A non-randomized trial conducted on healthcare workers found that SARS-CoV-2 spike-specific memory B and T cells were still detectable 5 months after the second dose of the inactivated vaccination, which may form the basis of a rapid recall response to the booster dose of the vaccine (18). As observed in healthy individuals, a rapid and robust recall of immune responses following the booster dose of the inactivated vaccine may also exist in PLWH.

Our study reveals that the booster dose vaccination among PLWH reduced antibody decay maintained a higher nAb titer and sustained T cell responses even 6 months after vaccination. The limited literature on this supports our findings, as the previous study indicated that in healthy individuals who received three doses of the inactivated or mRNA COVID-19 vaccine, nAb titers decayed much slower within 6 months after booster dose administration than after primary two-dose regimens and still maintained a higher level 6 months after the booster dose vaccination (19, 20). It is well known that immune memory leads to the maintenance of immunity. The booster doses of both the inactivated and mRNA vaccine were reported to elicit extensive expansion of memory pools, especially robust SARS-CoV-2-specific memory B cells, which may contribute to the prolonged duration of humoral and cellular immunity (18, 21). Taken together, our study confirms the efficacy of the booster dose of the inactivated vaccine among PLWH for not only dramatically enhancing but also prolonging protective immunity against SARS-CoV-2 infection.

Lower immunogenicity against the booster dose of the vaccine was found at multiple time points among PLWH than among healthy individuals. These findings are consistent with immunogenicity toward the first and second doses of the COVID-19 vaccine. For example, previous studies reported that the humoral and cellular immune responses against two doses of the inactivated COVID-19 vaccine were lower, and the antibody responses were more delayed among PLWH than among healthy individuals (7, 22, 23). Our study supplements the comparison of the humoral and cellular immune responses against the booster dose vaccination between PLWH and HC, suggesting that the immunogenicity of the booster dose of the vaccine among PLWH is still lower than among healthy individuals. Lower immunogenicity in PLWH compared with HC has also been reported in other vaccine, such as the inactivated influenza and pneumococcal conjugate (24, 25). Previous studies indicated that PLWH with a CD4 count of less than 200 cells/μl had significantly weakened immune responses to COVID-19 vaccine compared with healthy individuals (26, 27). In our study, 95.4% of PLWH had a CD4 count over 200 cells/μl, most even had a CD4 count over 500 cells/μl. However, we still observed decreased immunogenicity to vaccine among PLWH compared to among HC. Chronic immune activation and immune dysregulation induced by HIV infection lead to immune damage of B cells and T cells, which may be related to lower immunogenicity against vaccine among PLWH (28, 29).

Our study has several limitations. First, the sample sizes of the PLWH and HC enrolled are relatively small; however, they are comparable to or larger than those of previously published studies (30, 31). Furthermore, significantly lower immunogenicity against the booster dose of the vaccine among PLWH than among HC was observed despite the limited sample size. Second, the time points for the humoral immunity assessment were not completely consistent between the second and third doses of the vaccine, but we demonstrated the antibody response differences between PLWH and HC using the same follow-up time points. Third, *in vitro* assays were not conducted to further investigate the efficacy of three doses of the inactivated vaccine against the variants. More studies are needed to assess the protection of the inactivated vaccine against the SARS-CoV-2 variants of concern among PLWH.

## Conclusions

Among PLWH, the booster dose of the inactivated vaccine could induce stronger nAbs levels, sustain T cell immunity, and reduce antibody decay. However, the antibody and T cell responses against the booster dose of the inactivated vaccine were lower among PLWH than among healthy controls. Our study supports the efficacy of a booster dose of the inactivated vaccine in PLWH. However, additional strategies are needed to improve the immune response to the inactivated COVID-19 vaccine in PLWH.

## Data availability statement

The original contributions presented in the study are included in the article/**Supplementary Material**. Further inquiries can be directed to the corresponding authors.

## Ethics statement

The studies involving human participants were reviewed and approved by the Research and Ethics Committee of Zhongnan Hospital, Wuhan University, P. R. China (2020079K-1). The patients/participants provided their written informed consent to participate in this study.

## Author contributions

KL and WT conceived and designed this investigation. YT, SZ, FM, and MW collected the original data. YT, SZ, FM, SW, and WG analyzed the data. YT and KL contributed to the writing of the paper. All authors read and approved the final manuscript.

## Supplementary material

Supplementary Figure 1 The gating strategy of the analysis of IFN-γ-secreting and TNF-α-secreting CD4^+^ and CD8^+^ T cells frequency in PLWH and HC.
