# Supplementary figures and images for: A tale of two conditions: when people living with HIV meet three doses of inactivated COVID-19 vaccines
Source: Front Immunol. 2023 Jun 19;14:1174379. doi: 10.3389/fimmu.2023.1174379 (PMC10315467; doi:10.3389/fimmu.2023.1174379)

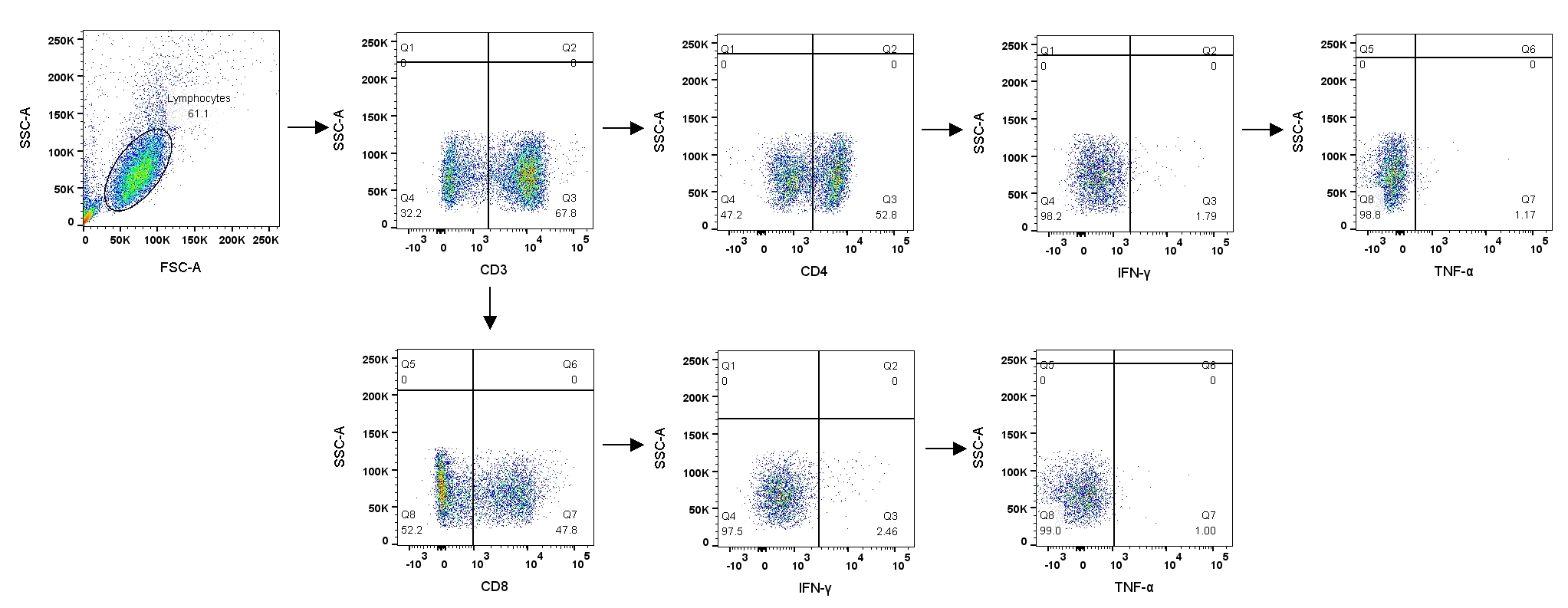

Supplement: Supplementary Figure 1 — The gating strategy of the analysis of IFN-γ-secreting and TNF-α-secreting CD4+ and CD8+ T cells frequency in PLWH and HC. [file Image_1.jpeg]
